# Expert consensus on the clinical strategies for orthodontic treatment with clear aligners

**DOI:** 10.1038/s41368-025-00350-2

**Published:** 2025-03-13

**Authors:** Yan Wang, Hu Long, Zhihe Zhao, Ding Bai, Xianglong Han, Jun Wang, Bing Fang, Zuolin Jin, Hong He, Yuxin Bai, Weiran Li, Min Hu, Yanheng Zhou, Hong Ai, Yuehua Liu, Yang Cao, Jun Lin, Huang Li, Jie Guo, Wenli Lai

**Affiliations:** 1 State Key Laboratory of Oral Diseases & National Center for Stomatology & National Clinical Research Center for Oral Diseases & Department of Orthodontics, West China Hospital of Stomatology, Sichuan University, Chengdu, China; 2 Department of Orthodontics, Ninth People’s Hospital Affiliated to School of Medicine, Shanghai Jiaotong University, Shanghai, China; 3 School of Stomatology, Department of Orthodontics, The Fourth Military Medical University, Xi’an, China; 4 State Key Laboratory of Oral & Maxillofacial Reconstruction and Regeneration, Key Laboratory of Oral Biomedicine Ministry of Education, Hubei Key Laboratory of Stomatology, School & Hospital of Stomatology, Wuhan University, Wuhan, China; 5 Department of Orthodontics, Beijing Stomatological Hospital, School of Stomatology, Capital Medical University, Beijing, China; 6 Department of Orthodontics, Peking University School and Hospital of Stomatology & National Center for Stomatology & National Clinical Research Center for Oral Diseases & National Engineering Research Center of Oral Biomaterials and Digital Medical Devices & Beijing Key Laboratory of Digital Stomatology & NHC Key Laboratory of Digital Stomatology & NMPA Key Laboratory for Dental Materials, Beijing, China; 7 Department of Orthodontics, School and Hospital of Stomatology, Jilin University, Changchun, China; 8 Beijing Tsinghua Changgung Hospital, School of Clinical Medicine, Tsinghua University, Beijing, China; 9 Department of Stomatology, The Third Affiliated Hospital of Sun Yat-sen University, Guangzhou, China; 10 Department of Orthodontics, Shanghai Stomatological Hospital & School of Stomatology, Shanghai Key Laboratory of Craniomaxillofacial Development and Diseases, Fudan University, Shanghai, China; 11 Hospital of Stomatology, Guanghua School of Stomatology, Sun Yat-sen University, Guangdong Provincial Key Laboratory of Stomatology, Guangzhou, China; 12 Department of Stomatology, The First Affiliated Hospital, College of Medicine, Zhejiang University, Hangzhou, China; 13 Nanjing Stomatological Hospital, Affiliated Hospital of Medical School, Nanjing University, Nanjing, China; 14 Department of Orthodontics, School and Hospital of Stomatology, Shandong University & Shandong Key Laboratory of Oral Tissue Regeneration & Shandong Engineering Laboratory for Dental Materials and Oral Tissue Regeneration & Shandong Provincial Clinical Research Center for Oral Diseases, Jinan, China

**Keywords:** Craniofacial orthodontics, Malocclusion

## Abstract

Clear aligner treatment is a novel technique in current orthodontic practice. Distinct from traditional fixed orthodontic appliances, clear aligners have different material features and biomechanical characteristics and treatment efficiencies, presenting new clinical challenges. Therefore, a comprehensive and systematic description of the key clinical aspects of clear aligner treatment is essential to enhance treatment efficacy and facilitate the advancement and wide adoption of this new technique. This expert consensus discusses case selection and grading of treatment difficulty, principle of clear aligner therapy, clinical procedures and potential complications, which are crucial to the clinical success of clear aligner treatment.

## Introduction

Malocclusion is a common oral disease with the estimated prevalence among general population ranging from 43.5% to 67.2%.^1,2^ It is associated with the risk of various oral dysfunctions and esthetic concerns, which may have detrimental effects on mental health and the quality of life.^3–6^ Recent years have witnessed the growing popularity of clear aligners among patients owing to their esthetic appeal, comfort, and convenience in oral hygiene maintenance.^7,8^ However, as a novel technology distinct from traditional fixed orthodontic appliances, clear aligner treatment (CAT) presents new challenges in case selection, treatment strategy, aligner design, and follow-up monitoring, which are associated with the differences in material characteristics and properties, and treatment outcomes.^9–12^ Therefore, key clinical aspects of CAT are demanded to help improve treatment efficacy and promote continued development and dissemination of this clinical technique.

Clear aligners are removable orthodontic appliances that were first introduced two decades ago and have been used to treat nearly 20 million patients worldwide. Since their launch, significant innovations have been achieved in the development of clear-aligned materials. The use of big data analyses and design software has enabled the aligners to tightly envelope the tooth surface and apply gentle continuous force which can be designed based on the desired tooth-specific movement direction and distance. The optimal sequence of tooth movement can be calculated precisely to ensure that tooth moves in the desired direction.^9,13^ Furthermore, clinical solutions have evolved from optimizing individual to optimizing group teeth movement, while clinical indications have expanded from simple to complex cases, including surgical cases.^14–16^

Consequently, CAT has become the primary innovating trend in orthodontics.^14,17^ To date, over 5000 publications on clear aligners have been indexed on PubMed, including case reports, clinical trials, retrospective clinical studies, and reviews, highlighting the on-going interest in this field.^18–22^ The purpose of this expert consensus was to summarize the core technology of CAT and provide clinical guidance for practitioners in terms of indications, treatment strategies, aligner design, and follow-up monitoring.

## Case selection and grading of treatment difficulty

### Indications and contraindications

The current indications for CAT are comparable to those for fixed orthodontics. Clear aligners can be used to treat nearly all types of malocclusions, especially the patients with high esthetic and comfort requirements, poor periodontal conditions, susceptibility to caries, or enamel developmental defects. However, clear aligners are not recommended for patients with clinically short crowns, requiring extensive mesial movement of the posterior teeth, or showing poor compliance.^20^

However, treatment difficulty of clear aligner therapy varies greatly among cases. Thus, we suggest difficulty-grading criteria for CAT.

### Grading of treatment difficulty

**Fig. 1:**
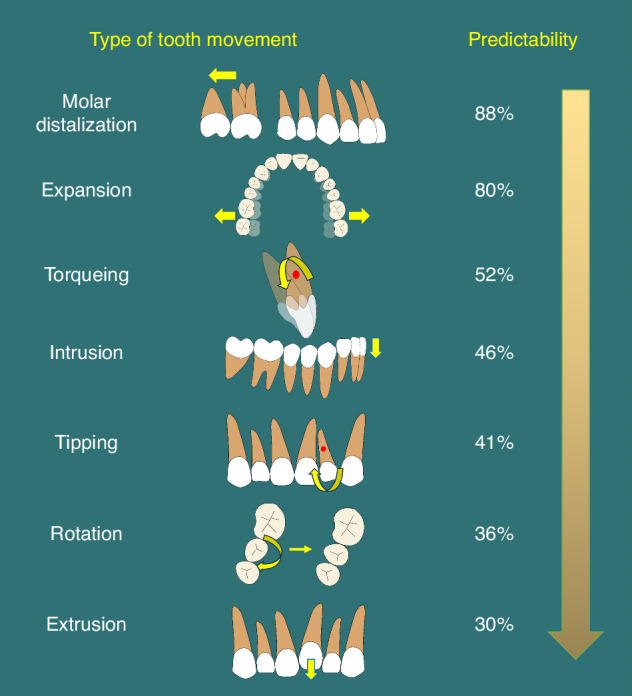
Predictability of different tooth movements achieved through clear aligner therapy

Clear aligners are made of elastic materials, and teeth are moved by the rebound force generated by the elastic deformations of the aligner materials when the aligners are positioned.^7^ Thus, aligners mainly provide a “pushing force”, and their clinical efficiency varies among different types of tooth movements (Fig. 1).^23–27^ Therefore, it is crucial to accurately assess treatment difficulty and select most suitable cases.

We developed the CAT-CAT difficulty assessment tool,^28^ which assigns scores based on model analysis, X-ray examination results, and clinical examination results. According to the literature and authors’ clinical experience, clinical cases were divided into four grades: easy, moderate, difficult and challenging (Table 1).

**Table 1:** Grading of clear aligner treatment difficulty

Difficulty level	Skeletal discrepancy	Crowding	Anterior overjet/overbite	Amount of molar movement	Associated manifestations	Treatment approach	CAT-CAT score
Easy	None	Mild crowding or spacing	Normal or slight abnormal	Molar distalization: <2 mm; mesial movement: 0 mm	None	Non-extraction; Arch expansion, IPR, or Mild incisor proclination	<20
Moderate	Mild	Moderate crowding	Deep overbite/overjet	Molar distalization: 2–3 mm; Mesial movement ≤2 mm	None	Molar distalization, Bite jumping, or Extraction of premolars	21–40
Difficult	Moderate to severe	Severe crowding	Severe deep overbite/overjet	Molar distalization >3 mm; Mesial movement >2 mm	Anterior open/crossbite; Posterior open/crossbite/scissor bite; Horizontal and/or vertical discrepancies	Extraction; Implant anchorage/elastics; orthognathic surgery	41–60
Challenging	Difficult cases with periodontal diseases, Temporomandibular Joint Disorders, missing molars, impacted teeth, molar protraction						>60

Owing to the biomechanical differences between CAT and traditional fixed orthodontics, it is imperative for clinicians to fully understand the characteristics of CAT and gradually implement treatment based on the difficulty level in each case to help minimize the associated risks.

## Principles of clear aligner therapy

Different from traditional fixed orthodontic appliances, clear aligners are made of elastic materials, which cover the whole or partial clinical crowns and create a “pushing” force produced from material deformation of the clear aligners. Thus, theoretically, the force can be designed to exert onto any part of the tooth crowns as long as it is closely covered by the aligners. Thus, the crowns’ surface area and the fitness of the aligners are the key points to the success of treatment. Attachments used in clear aligner treatment are bonded on the crowns, which can not only increase the surface area but also afford more action points of the force. Attachments in various shapes and sizes can be designed to supplement clear aligners for different biomechanical demands.

Besides, as we know, several types of arch wires made from different materials and in different shapes and/or sizes are used in traditional fixed treatment. In general, arch wires are used from thin to thick, round to rectangular, Niti to stainless steel, and therefore soft and flexible to solid and stable during the treatment. By doing so, teeth movement can be controlled in a predicted way. However, in clear aligner treatment, for each brand, the same aligner material is used throughout the whole aligner treatment, which is not as flexible as Niti wire nor as stable as stainless-steel wire. Thus, to move individual and/or group of teeth, tooth movement need to be designed in a stepwise mode, according to the natures of specific tooth movements.

**Fig. 2:**
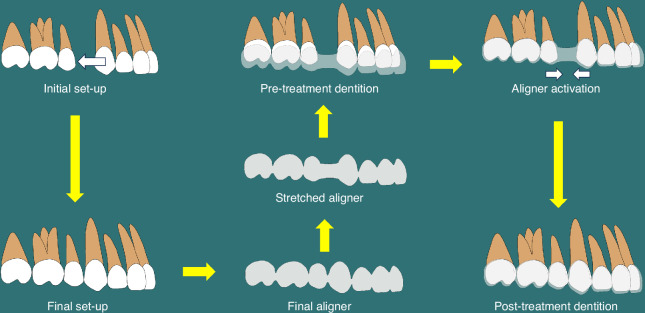
A schematic illustration of the principles of clear aligner therapy for incisor retraction in a premolar-extraction case. A final set-up is designed based on the initial set-up and a final aligner is fabricated based on the final tooth set-up. The final aligner is topologically distinct from the pre-treatment dentition and should be elastically stretched for being fitted onto the dentition. The stretched aligner on the pre-treatment dentition is activated and generate retraction force on the anterior teeth and protraction force on the posterior teeth, resulting in premolar-extraction space closure

Moreover, aligners’ elastic force is directly proportional to the amount of material deformation within a certain range, whereas excessive deformation can lead to plastic deformation, resulting in a loss of the force. Additionally, all the elastic force decreases with the deformation time.^9^ Therefore, when designing clear aligners, a series of intermediate statuses is used to bridge the initial and final status. The aligners are regularly replaced, helping the teeth move gradually to the desired position under the effect of a continuous gentle force (Fig. 2).

Thus, the initial, intermediate and final positions are the three keys to the success of clear aligner therapy. The initial position is determined based on patients’ characteristics, especially the digital dental models that capture the intraoral dentition and occlusion. Intermediate positions aim to ensure that the path and rate of tooth movement comply with the biological and biomechanical principles of orthodontic tooth movement. The ideal final position necessitates well-aligned dental arches, normal anterior overjet/overbite, and perfect posterior interdigitations.

Therefore, CAT is essentially a process of tooth repositioning in three dimensions. A critical aspect of this process is the acquisition and redistribution of space. There are currently five main methods for gaining space: arch expansion, molar distalization, incisor proclination, interproximal reduction (IPR), and extraction.^29–32^ Clinical treatment plans should be designed based on individual cases.

Next, we will discuss specific strategies for various clear aligner treatments in details, based on the methods of gaining space.

## Clinical procedures of clear aligner treatment

**Fig. 3:**
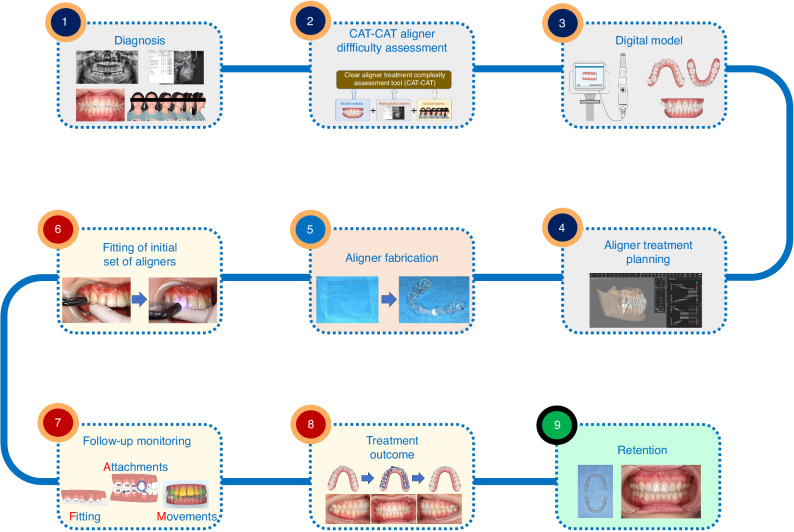
The overview procedures of clear aligner treatment

As illustrated in Fig. 3, clear aligner treatment encompasses nine procedures in clinical practice, starting from diagnosis, clear aligner treatment difficulty assessment based on CAT-CAT, acquisition of digital models and aligner treatment planning. Once the aligner treatment planning is ready, aligner fabrication ensues. Then, clear aligner treatment progresses to clinical section that involves fitting of initial set of aligners, follow-up appointments and monitoring, and end of the active clear aligner treatment. Lastly, retention is required and important following orthodontic treatment.

### Diagnosis

The precise initial position of the teeth requires complete and accurate patients’ data. And thus, data collection for CAT is essential, including facial and intraoral photographs, radiographic data [panoramic tomography, cephalometric radiographs, and cone beam computed tomography scans (CBCT)], and digital dental models that can be obtained through silicone rubber (PVS) impressions or intraoral scanning.^33,34^ Based on these patient data, a meticulous diagnosis is established.

### CAT-CAT aligner difficulty assessment

Orthodontic treatment goals are similar, regardless of treatment modalities. CAT plans should be based on patient complaints, presentation, and diagnosis. CAT can make orthodontic treatment easier, faster and more effective. However, before patients can be recommended for CAT treatment, difficulty level should be assessed (Table 1) to ensure patient suitability. And clinicians should ensure that they have made the correct diagnosis and appropriate treatment plans.^28^ As for some difficult or challenging cases, such as patients with severe periodontitis or needing surgical treatment, multi-disciplinary treatment (MDT) and specialists’ guidance are necessary.

### Digital models

As mentioned above, digital models can be acquired through either intraoral scanning or PVS impression taking.

### Aligner treatment planning

Recently, we developed a novel clear aligner treatment philosophy—biomechanics-guided, esthetics-driven, periodontium-supported and temporomandibular joint-compatible clear aligner therapy (BEPT-CAT)—that can guide practitioners to perform aligner treatment planning.^35^ Most cases of malocclusion are caused by “incorrect” tooth position, resulting in the discrepancies in necessary and available space. And thus, the treatment principles focus on either increasing the amount of space available or reducing the tooth amount. Common clinical methods for increasing the available space include arch expansion, molar distalization, and incisor proclination, while methods for reducing the tooth amount include IPR and extraction.^36,37^

#### Arch expansion

##### Indications

- Narrow dental arch: a narrow dental arch can be determined based on the relationship between the most prominent points on the buccal surfaces of the crowns of the lower posterior teeth and the Wala ridge.^38^ Pont index analysis and Howes value can also assist in the width assessment.^39^ Pretreatment CBCT can be used to clarify the spatial relationship between the root and alveolar bone, which helps avoid excessive expansion that may result in bone fenestration or dehiscence.
- Excessive buccal corridor: excessive buccal corridor refers to excess negative space between the dental arch and the buccal mucosa of the oral cavity. Previous studies have shown that an excessive or insufficient buccal corridor jeopardizes smile esthetics.^40,41^ An excessive buccal corridor is indicative of the arch expansion.

##### Considerations for final position design

**Fig. 4:**
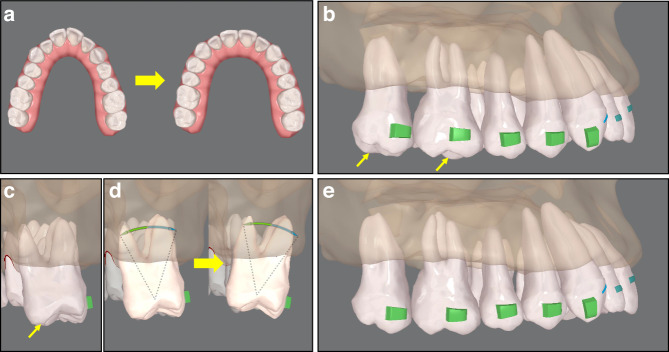
Aligner treatment planning for arch expansion. **a** A narrow upper arch is to be expanded. **b** From the buccal view, the palatal cusps of the molars can be observed (yellow arrows). **c** From the posterior view, the palatal cusp (yellow arrow) is more occlusal than the buccal cusps. **d** Buccolingual angulation of the molar is modified and a buccal root-torque is added. **e** The palatal cusps cannot be observed from the buccal view

Factors that must be considered include arch symmetry, arch coordination, and appropriate expansion amount to prevent bone fenestration or dehiscence. The volume of basal bone on buccal side should be analyzed in CBCT to determine the upper limit of the expansion. The amount of up-to-2 mm expansion on each side is safe in most cases. As for adolescents, the greater regenerative potential of alveolar bone remodeling makes arch expansion much safer. To prevent buccal inclination of crowns during expansion, the final position design should ensure that all the expanded posterior teeth are in lingual inclination (from the lateral view, the palatal cusps are invisible) (Fig. 4).^42^

##### Attachment design

Attachments are required on the buccal surfaces of teeth during arch expansion to prevent buccal inclination. For teeth with inadequate height of lingual cusps, lingual attachments may be placed simultaneously.^43^

##### Considerations for staging

It is recommended to design a staged expansion for any expansion exceeding 1 mm unilaterally, such as a “V-pattern” design like molar distalization. Homonymous teeth in the same jaw are suggested to expand simultaneously because they can act as reciprocal anchorages.

By adhering to these principles, clinicians can effectively incorporate arch expansion into clear alignment treatment plans, ensuring optimal outcomes in patients with dental arch discrepancies.

#### Molar distalization

##### Indications

- Almost normal facial pattern with distal (Class II) or mesial (Class III) molar relationship may be an indication for molar distalization. It may be accompanied by mild to moderate crowding, deep overjet, or an anterior crossbite/edge-to-edge bite. However, molar distalization is not generally recommended for neutral molar relationship (Class I).^44,45^
- Sufficient space in the posterior dental arch is necessary for molar distalization. CBCT evaluation from a three-dimensional perspective is recommended for molar distalization greater than 2 mm. Vertically, the presence of a low maxillary sinus increases the difficulty of upper molar distalization, especially when the molar roots penetrate the cavity. Third molar extraction is recommended to reduce distalization resistance and provide more space.^44,46^

##### Considerations for final position design

The upper limit of molar distalization of clear aligner treatment depends on the available retromolar space. The third molars can be extracted if there is no sufficient space. The amount of less than 2 mm molar distalization on one side is considered predictable in most cases while the mesio-distal inclination of posterior teeth and the potential of bone growth in children and adolescents should be taken into consideration.

Based on the literature and clinical experience, the predictability of molar distalization using clear aligners is approximately 88%.^23^ Thus, it is feasible to design the final position based on the actually required distalization distance (i.e., to obtain a neutral relationship) where no or minimal overtreatment is required. Additionally, to prevent labial fenestration and/or dehiscence in the lower anterior region, it is necessary to avoid labial movement of the lower anterior teeth, particularly the roots. This is because class II intermaxillary elastics are commonly applied during upper molar distalization, which exert a mesial force on the lower arch and labially push the lower anterior teeth.^47^

##### Attachment design

Molar distalization does not require the supplement of attachments. However, attachments are recommended to enhance the grip of teeth with short crowns. Moreover, molar distalization is often accompanied by other complex movements such as intrusion and rotation, and attachments are usually required to improve the success rates of these movements and prevent off-tracking. Traditional rectangular attachments are generally designed for the canines to increase the retention of aligners and minimize the impact of precision cuts.^48–50^

##### Intermaxillary elastics

When clear aligners exert a pushing force to achieve molar distalization via material deformation, the counteracting force may procline the anterior teeth. Thus, if anterior tooth proclination is undesirable, the anchorage of the anterior teeth should be reinforced. Intermaxillary elastics are commonly used in practice to achieve this aim.^45^

**Fig. 5:**
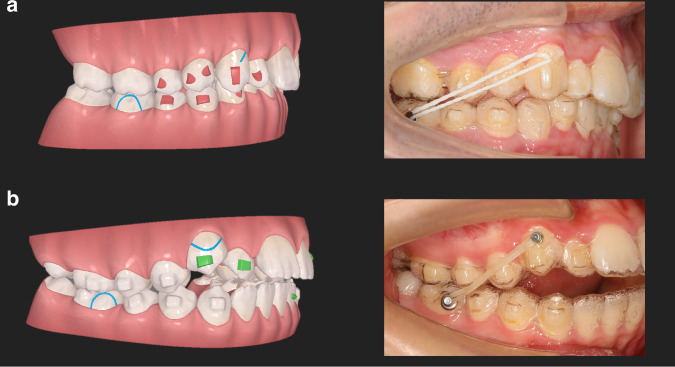
Different modes of elastic tractions. **a** Class II elastic traction is applied on the precision cut on the upper aligner. **b** A cut-out is designed on the upper canine and class II elastic traction is engaged ono the button on the canine

In maxillary molar distalization, precision cuts are designed at the maxillary canines, whereas buttons are bonded to the buccal surface of the mandibular first molars (cut out on lower aligners) to allow the use of Class II intermaxillary elastics (Fig. 5a).^51^ If simultaneous eruption of the canine is desirable (e.g., low positioned or insufficiently erupted canines), a button can be bonded to the labial surface of canine near the gingival margin to facilitate eruption (Fig. 5b). However, precision cuts at the mandibular molars are prone to aligner displacement or off-tracking and are not recommended. Additionally, if necessary, implant devices can be used to enhance the anchorage, provided they do not obstruct molar distalization.^52–54^ On the other hand, if the proclination of anterior teeth is desirable (e.g., Class II Division 2), it can be designed simultaneously with molar distalization, acting as reciprocal anchorage to eliminate the need for any elastics.^55^ Nevertheless, anterior proclination and molar distalization should be closely monitored during follow-up appointments for real-time adjustments.

##### Considerations for staging

**Fig. 6:**
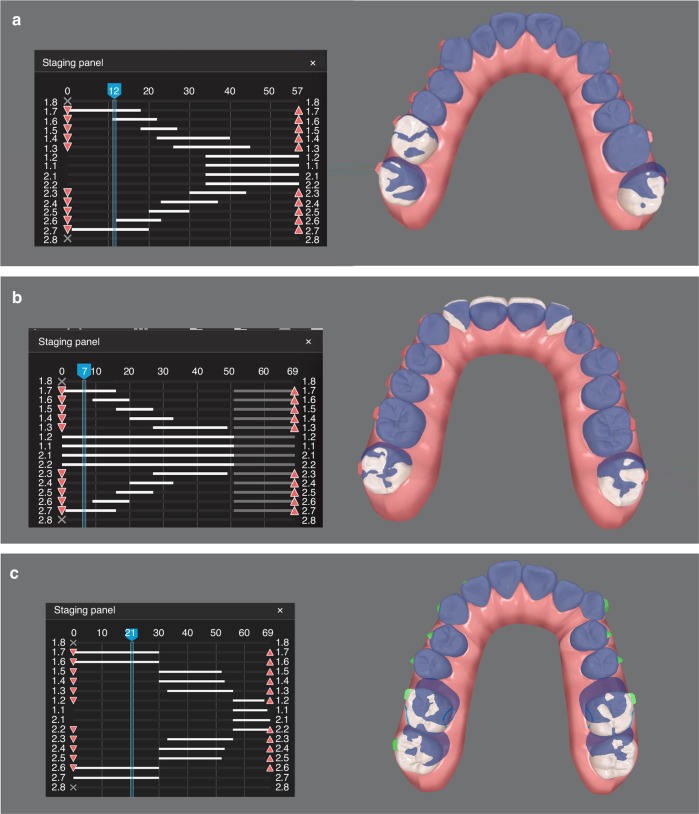
Different aligner design patterns for molar distalization. **a** Strict V-pattern. Molars, premolars and anterior teeth move sequentially. **b** Modified V-pattern. Incisors move alongside molar distalization. **c** The first and second molars move simultaneously

The staging of tooth movements involves the consideration of anchorage. Typically, molar distalization is designed in a “V-patten” staging, in which the second molars are moved first, and then the first molars once the second molars have reached the halfway point of their total moving distance; thereafter, the second premolars start to move once the second molars have completed their “journey” (Fig. 6a). Thus, no more than four teeth are distalized at each stage (V-pattern).^56^ Finally, the space created by canine distalization can be used to align and/or retract the anterior teeth. By doing so, the anchorage is often adequate for most distalization cases; however, a long-term treatment is unavoidable.^48^ In some cases, in order to shorten the treatment duration and increase patient compliance and cooperation, alignment of the anterior teeth is performed simultaneously with molar distalization, allowing patients to observe quick esthetic changes (Fig. 6b). In addition, implant screws can be used to strengthen anchorage, allowing more teeth to distalize simultaneously, to shorten treatment duration (Fig. 6c).^57–59^

#### Proclination of anterior teeth

##### Indications

Patients presenting with straight or concave facial profiles and retro-inclined or upright anterior teeth accompanied by mild crowding, such as cases with deep overbite caused by lingual inclination of the upper anterior teeth, are indicated for proclination of anterior teeth, which can be combined with other methods to obtain enough space.

##### Considerations for final position design

The sagittal position and proclination of the anterior teeth, especially the upper anterior teeth, are crucial for facial esthetics and are one of the main indicators for profile analysis.^60–63^ Thus, the degree of proclination of the anterior teeth should be carefully evaluated based on facial morphology, and a combination with other methods that help acquire sufficient space should be considered. For patients with a severe lingually inclined deep anterior overbite, the roots-and-bone relationship should be considered. The roots need to be positioned within the cancellous region of the alveolar bone.^64,65^ Theoretically, a proclination of 1 mm (2.5°) in the anterior segment provides 2 mm of space. Therefore, the proclination design in the final position is based on the amount of space required, facial morphology, and the roots-and-bone relationship.^66^

##### Attachment design

More than 3° of incisor proclination activates the power ridge in the designing software system, which applies labial-torquing force on the crowns, whereas lingual-torquing force on the roots and effectively achieves root-controlled movement of the anterior teeth.^67^ Traditional attachments on canines are recommended to reduce the risk of aligner off-tracking in the anterior segment.

##### Considerations for staging

A minor proclination can be synchronized with the alignment of mild crowding. However, in cases with lingually inclined deep overbite, staged tooth movement is required. Proclination is first performed to torque the roots into the cancellous bone, and then followed by intrusion and retraction of the anterior teeth.

#### Interproximal reduction (IPR)

##### Indications

Although IPR is a method for gaining space, it has always been controversial because of the potential damage to the enamel and the resulting risk of caries. The authors suggested that IPR should be used as a supplement to other methods, rather than as the primary method, to gain space. The following situations warrant an IPR design^29,31^:

- Bolton discrepancy due to the missing teeth or malformed teeth.
- Gingival embrasure defects (black triangles) due to periodontal disease.
- Poor crown morphology with contact points nearby the incisal edge.

##### Considerations for final position design

In general, IPR is designed in the anterior segment, if needed. It is advisable to limit the maximum amount of IPR to 0.25 mm on the proximal surface of each tooth. Studies have shown that IPR amounting to no more than 50% of the enamel thickness generally does not increase the risk of caries.^68–70^

##### Considerations for staging

Since the IPR site is the anatomical contact point of the crown rather than the actual contact point, restoring normal contact points first undoubtedly facilitates IPR performance. However, in practice, there may be situations in which insufficient space hinders the alignment of the dental arch, which requires a comprehensive assessment of the timing of IPR. Graded IPR is recommended to alleviate this contradiction. Fluoride application after IPR performance is suggested.

#### Tooth extraction

Tooth extraction is a common method for reducing tooth amount in orthodontic treatment and is mainly indicated when the discrepancy between the available and required space exceeds 8 mm, such as in cases with severe crowding or severe maxillary and/or mandibular protrusions. Two types of tooth extraction patterns are commonly used in clear alignment treatment: extraction of lower incisor and extraction of premolars (first or second).

#### Extraction of lower incisors

##### Indications

- An almost normal facial pattern with stable posterior occlusion, no indication for upper extraction, and the total required space in the mandible exceeding 6 mm.
- Bolton ratio discrepancy due to missing teeth or malformed teeth in maxilla.
- Poor prognosis of a lower incisor due to periodontal disease or dental trauma.

Considerations for final position design: The extraction of a lower incisor results in the lack of the midline of the lower dental arch. Instead, the long axis of the lower central incisor may be designed as the lower midline. In most cases, IPR of the upper anterior teeth is necessary to resolve the discrepant Bolton ratio and achieve normal anterior overbite and overjet.^71^

Attachment design: it is recommended to design vertical rectangular attachments or root-control attachments on the adjacent teeth to the extraction space, which facilitate the reciprocal movement of the adjacent teeth, especially their roots.^72^

Considerations for staging: extracting a lower incisor can effectively relieve crowding in the lower anterior section and provide space for the intrusion of the lower anterior teeth, resulting in a high rate of treatment success. Therefore, special staging considerations are generally not required.

##### Extraction of the first premolars

Based on the symmetry principle, the extraction of the first 4 premolars is the most common pattern of extraction in orthodontic practice. However, cases needing the extraction of 4 premolars belong to difficult level in CAT (Table 1), and clinicians need to reach a certain level of orthodontic experience to complete the treatment.

Indications: Extraction of the first 4 premolars is indicated when the discrepancy between the available and required space exceeds 8 mm, such as cases with severe crowding and/or bimaxillary protrusion, and etc.^73^

Considerations for final position design: most cases with tooth extraction are challenging to treat, as extensive tooth movement is unavoidable, requiring three-dimensional repositioning of these teeth. Treatment success relies on the torque control of the anterior teeth and the mesial-tipping avoidance of the posterior teeth.^74–76^ Therefore, the final position requires an over-treatment design, as follows:

- Anterior teeth exhibit a labial inclination with incisor angles of approximately 120°. To prevent excessive lingual inclination, adequate labial inclination and torque control (root-lingual torque) should be designed during the whole procedure of anterior retraction.^77^ Cases with more lingual inclination at the initial and/or longer retraction distances require a larger positive torque in the design.^78^
- Anterior teeth are in a shallow overjet/overbite or edge-to-edge position without occlusal contact. The pendulum effect of anterior retraction, compounded by any pre-existing deep bite condition, may require the over-treatment of anterior intrusion.
- Canines are mesially tipped with the roots closer to the extraction space.
- Posterior teeth are distally tipped, with additional negative torque to prevent buccal inclination of molars and loss of posterior anchorage.^79^

Attachment design: In such cases, attachment design should consider the following:

- Power ridge on incisors is recommended to aid in the torque control of the anterior teeth, which can be activated when more than 3° root-lingual torque is designed.
- Optimized attachments with strong root control or traditional rectangular attachments are recommended for the canines.^74^
- Horizontal rectangular attachments with strong retention are recommended for posterior teeth.

**Fig. 7:**
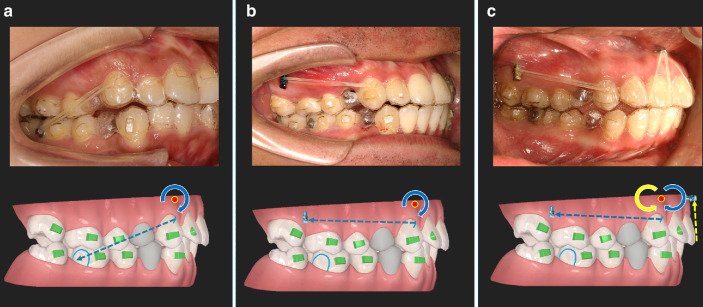
Different elastic tractions and biomechanical features. **a** A class II elastic traction is applied between the precision cut on the upper canine and the button on the lower first molar. Since the traction force (blue dashed line) passes occlusally to the center of resistance (red dot) of the upper anterior teeth, a clockwise moment (blue curved arrow) is generated. **b** An elastic traction is engaged between the precision cut on the upper canine and a buccal mini-implant. Likewise, a smaller clockwise moment (blue curved arrow) is generated. **c** One elastic traction is applied between the precision cut on the canine and a buccal mini-implant and the other one is engaged between the aligners on the incisors and a labial mini-implant. The retraction force (blue dashed line) generates a clockwise moment (blue curved arrow) while the intrusion force (yellow dashed line) offers a counterclockwise moment (yellow curved arrow). The two moments are offset by each other and the anterior teeth are retraction in a bodily movement mode

Intermaxillary elastics: To increase posterior anchorage, Class II elastics can be designed during anterior retraction (precision cuts at the upper canines and bonding of buttons on the buccal surface of the lower first molars). Alternatively, implant anchorage can be used in the anterior region to assist the intrusion and body retraction of the anterior teeth.^80–82^ Different modes of elastic tractions with or without mini-implants and their corresponding biomechanics are displayed in Fig. 7.

Considerations for staging: a personalized design is suggested for each case. The staging design should vary according to the specific circumstances because of the complex and variable nature of extraction cases. However, in most cases, we recommend distalizing canines and distal tipping of the posterior teeth (anchorage preparation) first. When canines complete the first third of the total moving distance, 6 anterior teeth start to move simultaneously by then. And finally, mesial movement of the posterior teeth begins when anterior teeth movement is completed. To prevent the “bowing effect”, it is suggested to avoid mesial movement of the posterior teeth simultaneously with the retraction of anterior teeth.

##### Extraction of the second premolars

Indications: In the following cases, second premolars are extracted instead of first premolars, which usually increases the treatment difficulty. Clinicians should be cautious to make a treatment scheme design like this:

- Serious damage/abnormality on the second premolar and/or its periodontal tissue.
- Second premolar is impacted or blocked-out of the dental arch.
- Minimal anchorage design.

Considerations for final position design: Compared to those in the first premolar extracted case, molars should be designed with more distal inclination (anchorage preparation) since the molars are more prone to mesial tipping, especially in the cases that more than 3 mm mesial movement of molars is required (minimal anchorage design), while less over-treatment of anterior teeth is needed.

Considerations for staging: we suggest, firstly, a sequential distal movement of the first premolars and canines, and distal-tipping anchorage preparation of the first molars. Then, move anterior teeth afterwards. And finally, mesially move the molars sequentially.

##### Bite jump (surgical and growth jump)

A bite jump refers to the changes in the three-dimensional position of the mandible and/or mandibular dental arch resulting from intermaxillary elastics, self-growth, and/or orthognathic surgery. It is important to note that the design of bite jump should be tailored based on the specific circumstances of the patient, and clinical feasibility should be considered. Except orthognathic surgery, bite jumps caused by other methods develop gradually in clinical practice, which can span the whole course of treatment.

Indications:

- Adolescents with mild skeletal or functional mandibular hypoplasia or retrognathia;^83,84^
- Functional Class III, with the mandible being able to retrude to edge-to-edge occlusion;
- Severe skeletal deformities requiring orthodontic-orthognathic treatment^22^;
- Mandibular malposition caused by premature individual tooth contacts.

Intermaxillary elastics: The sagittal bite jump requires the use of intermaxillary elastics or orthodontic appliances with mandibular advancement function.^85,86^

Considerations for staging: In the design software, bite jump can be placed at any stage of the treatment or throughout the treatment process. The authors typically place bite jump at the end of the treatment, which makes it easier for clinicians to assess the amount and direction of the jump and detect any abnormalities in a timely manner during clinical monitoring.

Below, we are going to delve into some special considerations in clear aligner design. A lot of clinicians are confused by these issues in practice.

##### Special considerations in clear aligner design

Over-treatment design: as we discussed before, clear aligners exert mainly a “pushing force”, and therefore their clinical efficiency varies among different types of teeth movements (Fig. 1). To better realize the actual teeth movement, over-treatment design is recommended in some cases, which is related to the predictability of CAT. For example, to intrude anterior teeth and correct deep bite, a shallow overbite and even open bite is designed in the final position, while large positive torque may be given to the incisors which are lingual inclined or up-righted initially when retraction of anterior teeth is required to correct the convex profile. However, the appropriate amount of over-treatment design is determined case by case, and until now, there is no consensus on this specific issue. According to our experience and previous clinical studies, the amount of over-treatment should be designed based on the initial status of teeth and the type and amount of the teeth movement.^78,79^

Challenges and strategies in the complex tooth movements: compared to expansion and molar distalization, intrusion, extrusion and torque control are more complex tooth movements in CAT, which have much lower predictability (Fig. 1). Thus, over-treatment is commonly designed for these types of movements.

Besides, sufficient space for tooth movements should be taken into considerations. For intrusion, the root-and-bone relationship needs to be analyzed in CBCT images to make sure that the roots are in the cancellous bones, while for extrusion, the intermaxillary space is required. And loose proximal contact points are always good for the movement.

**Fig. 8:**
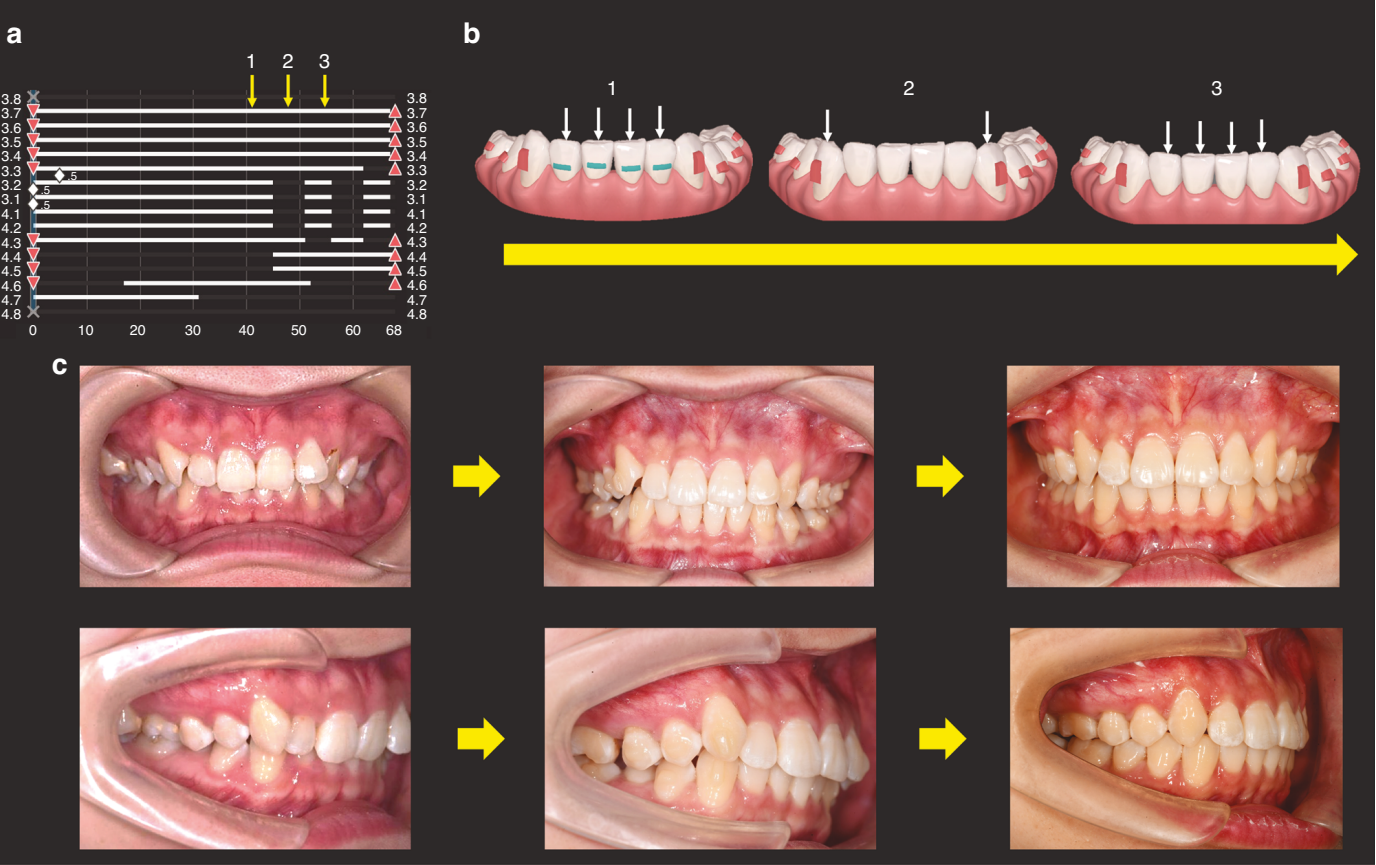
Staging design for the intrusion of anterior teeth in the lower arch. **a** Incisors and canines are intruded in an alternate mode. Incisors are intruded first (referred to stage 1), followed by the intrusion of canines (referred to stage 2). Then, incisors are further intruded (referred to stage 3). **b** Lower arch models showing intrusion of canines and incisors corresponding to the aforementioned three stages. **c** Anterior and side views showing the successful intrusion of the anterior teeth

Then, sufficient anchorage for the movement is important. There are usually two ways to strengthen anchorage in CAT. One is to move teeth in a stepwise mode. We recommend a “Frog pattern” staging for anterior teeth intrusion, in which incisors and canines are intruded separately and in cycles (Fig. 8). Extrusion of posterior teeth is suggested to be designed in a “V pattern” staging. Besides, power ridge design and positive torque is distributed in the whole procedure of incisor retracting to provide a better torque control. The other way to enhance anchorage is to use auxiliary devices and elastics, such as temporary anchorage devices (TAD) implanted in the anterior section to provide an extra intruding force and root lingual torque on anterior teeth (Fig. 7).

Furthermore, appropriate attachment design could provide clear aligners with greater retention, which is the key for CAT. Traditional attachments on premolars are recommended when intrusion of anterior teeth is needed while traditional attachments on canines are suggested for incisor’s torque control.

Differences in the design of CAT between adolescents and adults: as we know, the main difference between adolescents and adults is growth potential which may lead to different orthodontic treatment plan. Mandible growth can result in anteroposterior bite jump, and thus, bite jump design without surgery is more possible to realize in adolescents. Besides, the prevalence of oral caries is higher in adolescents, and therefore, interproximal reduction (IPR) design should be used more cautiously. Moreover, traditional attachments or optimized attachments in larger size are recommended in adolescents due to their inadequate crowns. A recently published expert consensus on adolescents’ orthodontic treatment has deeply discussed this special issue.^6^

### Aligner fabrication

Once aligner treatment planning is ready, clear aligners that move teeth incrementally can be fabricated based on either thermoforming or 3D printing.

### Fitting of initial set of aligners

Patients are informed to the clinic for the initial appliance placement when clinicians receive the aligners. On this day, the resin attachments are bonded onto the teeth according to the digital design, and the first set of aligners is tried in (fitness should be checked). Subsequently, patients are issued a set of instructions, including the required wearing duration, method of aligner placement, and usage of chewies. The patients are also informed about the importance of oral hygiene. Additional information and instructions are provided to the patients, as relevant, depending on the tooth movement plan, such as molar distalization, IPR, or extraction.

### Follow-up monitoring

#### Patient compliance management

Regular follow-up visits are essential and can be used to inform patients about treatment progress and challenges, helping them understand their roles in the process, increasing their confidence, compliance, and cooperation.^18,87^

Cooperation in the long duration of orthodontic treatment is a huge challenge to majority of people, especially persisting in wearing clear aligners day by day. Thus, close contact with patients helps to know their status and give them a hand or timely reminding if needed. Pleasure communication and compliments on patients are always effective in maintaining good relationship between clinicians and patients, which is beneficial for the cooperation as well. To encourage patients, practitioners can show them the changes already occurred by comparing with their pre-treatment photos and inform them that all these changes are owing to their compliance and cooperation. Let patients be aware of that their efforts will pay back. By doing so, patients will be more confident in the treatment.

Besides, some application programs registered by patients’ ID number can be used on smart cell phone to help record the wearing date and remind to change a new set, which is convenient for patients in daily life.

#### Things to do in the follow-up visits

To evaluate treatment progress, comprehensive examinations should be performed, including the following assessment:

- Tooth and periodontium status assessment, including mobility, premature contact presence, and occlusal trauma.^88,89^
- Occlusion changes, including the sagittal relationship, occlusal contacts, inclination, midline of upper/lower dental arch, overjet, overbite, torque and space, comparing to baseline and digital design.
- Temporomandibular joint health assessment should interrogate any pain, tenderness, and clicking in the joint area, especially in patients with temporomandibular disease before treatment and in adult patients using intermaxillary elastics.^90–93^
- Any detachment and/or abrasion of attachments should be checked according to digital design.^94^
- Aligner fitness assessments account for the progress in tooth movement, especially any gap observed in the space from the incisal edges of the anterior teeth, cusps of the posterior teeth, and the area around the attachments and along the aligner margin.

#### Management of off-tracking

**Fig. 9:**
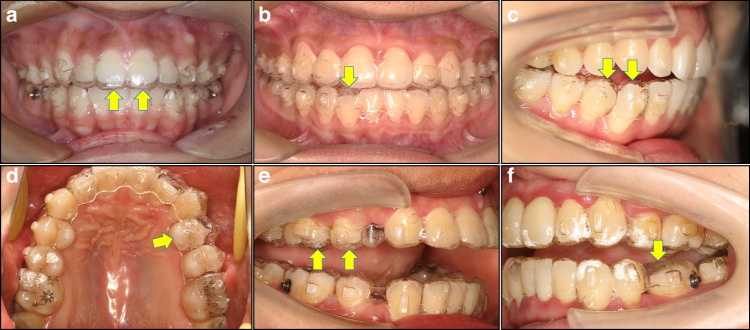
Aligner off-tracking. **a** Off-tracking of upper incisors (yellow arrows). **b** Off-tracking of a lower incisor (yellow arrow). **c** Off-tracking of a lower canine and first premolar (yellow arrows). **d** Off-tracking of an upper premolar (yellow arrow). **e** Off-tracking of an upper premolar and molar (yellow arrows). **f** Off-tracking of a lower molar (yellow arrow)

**Fig. 10:**
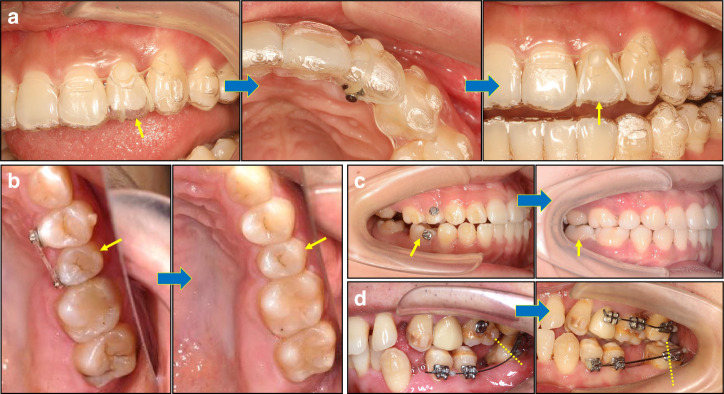
Strategies for resolution of tooth off-tracking. **a** A “boot-strapping” technique was used to address the off-tracking of a lateral incisor by applying labial-lingual elastic traction. **b** A power chain was used to correct under-derotation of a second premolar. **c** A vertical elastic traction from the upper premolar to the lower molar was implemented to correct mesial tipping and intrusion of the lower first molar. **d** A segmental archwire technique was applied to correct mesial tipping of a lower second molar

Off-tracking refers to the incomplete fitting between the teeth and aligners, indicative of a discrepancy between the direction and/or distance of actual tooth movement and that planned in the digital design (Fig. 9). The management of off-tracking involves removing attachments and using aligners to guide the off-tracking teeth back into the desired path using intra-/inter-maxillary elastics. Off-tracking manifestations can be categorized into the following three situations:

- Off-tracking in the vertical dimension due to insufficient extrusion or anterior intrusion.^95^ Insufficient extrusion may manifest as uniform vacuoles emerging at the incisal edges or cusps and can be managed by removing the attachments on the off-tracking teeth and applying intra-/inter-maxillary elastics (Fig. 10a). Alternatively, in cases of insufficient anterior intrusion, which manifest as inadequate correction of the anterior deep bite, auxiliary devices, such as implants or redesigning additional aligners to increase the staging design for tooth movement, may be added.
- Off-tracking in the horizontal dimension commonly occurs in rotation correction, especially in severely rotated premolars.^96^ The removal of attachments and use of a power chain can be helpful in most cases (Fig. 10b).
- Off-tracking in the sagittal dimension is characterized by mesial inclination of the posterior teeth and torque loss of the anterior teeth (lingual inclination).^75,76^ Mismatches between the attachments and vacuoles on the aligners can be observed on mesially inclined posterior teeth, as well as the gaps between the mesial cups and aligners. Distal up-righting of these off-tracking teeth must be performed using intermaxillary elastics and/or sectional arch wires after the removal of the attachments (Fig. 10c, d).
- The loss of anterior tooth torque manifests as lingual inclination of the upper/lower anterior teeth, increased overbite, early contact of anterior teeth, and posterior open bite.^97^ In such cases, the aligners may need to be redesigned to restart the program.

#### Timing and considerations of program restart

Sometimes not only one series of clear aligners are needed to complete the treatment. There are five possible reasons for this:

- The discrepancy between designed tooth movement and actual tooth movement, which result in an incomplete correction of the malocclusion, often occurring in some complex tooth movement, like intrusion, root control and more than 3 mm molar distalization. More series of aligners are designed to accomplish the treatment goal.
- Unwanted tooth movement occurs and leads to reduced occlusal contacts or even open bite in posterior segment, which may be due to the aligners’ effect of occlusal pad. More series of aligners are designed to consolidate the occlusion.
- More teeth should be included into treatment, which is common in adolescents with erupting second molars. A new series of aligners are usually designed to cover these second molars and some heterotopic or impacted teeth, if any.
- The change of occlusal relationship may occur, due to mandible growth and/or removal of occlusal interference. Then, a completely new design should be done according to the new and stable occlusal relationship.
- Bad cooperation in patients, leads to serious off-tracking, and even totally unfitting. A new series of aligners are designed based on current status.

### Treatment outcome

Treatment is complete after waring the final set of aligners, if the treatment objective has been achieved. The criteria for ending CAT are consistent with those for ending traditional fixed orthodontic treatment. At the end of the treatment, the attachments and other auxiliary devices are removed, and retainers are prescribed as usual.

### Retention

Retention is of vital importance to clear aligner treatment. Different modalities of retention can be chosen based on patient-specific characteristics, e.g., periodontal condition, caries vulnerability, etc. Patients should be recalled to check tooth alignment, retainer fitting, and signs of relapse.

## Complications

CAT is associated with some risks to dental and periodontal health.

### Caries

Poor oral hygiene during CAT can disrupt the oral microbiota, leading to white spot lesions or even caries.^98^ However, compared to patients undergoing fixed orthodontic treatment, patients wearing clear aligners have lower levels of white spot lesions, total bacterial plaque, and cariogenic bacteria in the saliva.^99–101^ This may be related to the reduced detrimental effect of clear aligners on oral hygiene.

### Root resorption

CAT may lead to root resorption. However, it reported that CAT applied a gentler force, resulting in a lower rate and severity of root resorption, compared to those observed in fixed orthodontic treatment.^102–105^ Factors such as post-treatment root position (relationship with the cortical bone), extraction, tooth position, and specific tooth movement patterns (intrusion and extrusion) are all risk factors for root resorption, whereas post-treatment root position is most closely related to root resorption.^106^ Therefore, reducing the risk of root resorption requires limiting root movement within cancellous bone and avoiding unnecessary reciprocal movement. Furthermore, a clear aligner design software with a root-bone system makes the root-bone relationship visible in the digital design, which helps reduce root resorption risks.

### Periodontal damage

Standard orthodontic treatments do not cause periodontal damage. However, orthodontic appliances may increase the difficulty of maintaining oral hygiene, leading to a higher rate of gingivitis and periodontitis. Clinical trials have shown that, compared to fixed orthodontic appliances, clear aligners are more favorable for maintaining periodontal health in patients.^107–110^ Moreover, for cases with an unsatisfactory periodontal status, design changes can help mitigate these risks by decreasing the speed of tooth movement, reducing teeth coverage by aligners, and prolonging the wearing duration for each set of aligners. Thus, clear aligners are recommended for patients susceptible to gingivitis and/or periodontitis.

Meanwhile, alveolar bone defect (fenestration and dehiscence) is also a common complication of orthodontic treatment. A recent study found that the incidence of fenestration in patients treated with clear aligner and fixed appliance was 23.96% and 26.18%, respectively.^111^ Another investigation also showed that non-extraction CAT was associated with increased presence of alveolar bone dehiscence and fenestration.^112^ Thus, root-bone relationship should be considered and evaluated carefully, especially arch expansion is designed.

### Relapse

After orthodontic treatment, relapse tends to occur because of incomplete remodeling of the periodontal tissues and muscular system. In the literature, relapse has been mainly linked to occlusal stability, types of tooth movement, root-bone relationships, and the balance of intraoral and extraoral muscle forces, with the type of orthodontic appliance used having minimal impact on relapse risk.^113–115^ The use of retainers and correction of oral bad habits (such as tongue-thrust swallowing, etc.) are currently considered the most effective measures for reducing relapse risk.

## Conclusion and expectation

The design of clear aligners continues to evolve, taking advantage of the novel materials and insights generated by global big data studies, leading to less difficulty in complex cases treatment, allowing more patients worldwide to achieve better treatment outcome by this technology. A novel clear aligner philosophy—biomechanics-guided, esthetics-driven, periodontium-supported and TMJ-compatible clear aligner therapy (BEPT-CAT)—may be applied in clinical practice to guide aligner treatment planning and execution. Moreover, the possibility of tiny attachments or attachment-free designs may become feasible, further improve patients’ comfort and esthetics during treatment. In the future, individual dental practices may be equipped with devices that allow to 3D-print the elements of the novel designs, further increasing treatment personalization.

Advances in science and technology are driving progress in orthodontics. Esthetic, comfortable, convenient, and efficient orthodontic treatment will be realized through digitally oriented invisible aligner technology, bringing CAT into mainstream use.
